# Yoga as Complementary Care for Young People Placed in Juvenile Institutions—A Study Plan

**DOI:** 10.3389/fpsyt.2021.575147

**Published:** 2021-06-04

**Authors:** Nóra Kerekes

**Affiliations:** Department of Health Sciences, University West, Trollhättan, Sweden

**Keywords:** adolescents, aggressive antisocial behavior, juvenile institutional care, mental health, substance use, yoga

## Abstract

Recent studies have established yoga practice as a mainstream complementary clinical tool within correctional environments. It is shown that regular yoga practice is coupled with improved impulse control, sustained attention, attenuated antisocial and self-harm behaviors, reduced stress, and psychological distress. No academic research until now has provided evidence of mental health benefits of yoga for institutionalized young people. In Sweden, each year more than thousand adolescents receive compulsory care at juvenile institutions run by the Swedish National Board of Institutional Care. These young people are characterized by substance abuse, aggressive and antisocial behaviors, high frequency of self-harm, and the experience of abuse. Most of them manifest attention problems, depression, anxiety, and impulsivity. They have a dramatically increased risk for recidivistic criminal behavior, continuous medical, and social care and untimely death. The present study plan aims at evaluating, with previously validated psychological measures, in a quasi-experimental design, the effects of yoga practice for institutionalized adolescents. Adolescents' experiences of participating in yoga practice will also be assessed by semi-structured individual interviews. Ethical approval was given by the Swedish Ethical Review Authority. It is hypothesized that yoga practice (in combination with the standard treatment within institutional care) will reduce institutionalized adolescents' aggression, antisocial behavior, anxiety, depression, and negative affect, and increase their cognitive flexibility (in the form of increased impulse control).

## Introduction

### Young People Placed in Juvenile Institutions

The Swedish National Board of Institutional Care (Statens institutionsstyrelse, SiS) runs special residential homes and provides individually tailored compulsory care for young people with psychosocial problems, substance abuse and aggressive antisocial behaviors. Care is provided under the terms of the Care of Young Persons (Special Provisions) Act (LVU) (1990:52) and the Secure Youth Care Act (LSU) (1998:603). The social services of the municipalities are responsible for handling and placing these young people into SiS, as well as taking care of their needs and support after their release from SiS. SiS runs 23 special residential homes in Sweden that currently holds about 700 adolescents in need of special, compulsory care (Personal communication with SiS statistic unit, January 2021).

Every year, the Social services commit over a thousand young people between 15 and 21 years old to SiS's juvenile institutional care due to drug abuse, criminal activity, and/or other socially destructive behaviors. According to SiS (1) the average length of special care in 2019 was 164 days per young person, amounting to ~206,000 care days a year. In 2019, Swedish municipalities alone spent 1.44 billion Swedish crowns (SEK) on young people's care committed to juvenile institutions.

Within this population of children and young adults, the majority has at least one but most often several coexisting psychiatric diagnoses (2), even though the primary reason for the compulsory care is their antisocial behavior, which hampers individual development. The most common diagnosis for both young males and females is Attention-Deficit Hyperactivity Disorder (ADHD), followed by Substance Use Disorder and Post-Traumatic Stress Disorder (PTSD). During the peculiar circumstances of the year 2020, due to the COVID-19 pandemic, a substantial proportion of Swedish adolescents reported more substance use (3) suggesting a possible increased pressure on SiS services in following years.

About half of the young people in the SiS juvenile institutional care report having experienced depression for a longer period of time, attention difficulties, and/or severe anxiety/tension. Almost every other female (47%) and more than 10% (13%) of males has attempted suicide. More than one in three has been the victim of physical and/or psychological abuse by a parental figure. About 40% report having witnessed violence against someone close to them, and nearly half of the females have been victims of sexual abuse (4).

A majority of the young people in SiS care have failed at school, have difficulties keeping up with the teaching, and/or have poor attendance. Their challenges are often complicated, and their environment (family relations, the circle of friends) has often had a substantial impact on their current situation.

The special residential homes run by SiS are the only treatment facilities in Sweden that have the right to forcibly detain individuals who have been taken into compulsory care according to the LVU Act. This right encompasses the options to place individuals under lock and key and/or physically restrain them if they pose a danger to themselves or others. In 2019, physical restraint was used 1,152 times in the residential homes run by SiS (1). According to SiS, most of these events involved a small number of young people and the common factor was a low functioning level, neuropsychiatric disorders, and comorbid psychiatric illness.

Recent research has shown that this population of adolescents has an increased need for improved and integrative treatments. A 3-year follow-up study of institutionalized young people found dramatically increased rates of criminal recidivism, use of inpatient care, and untimely death among young people in institutional care compared to an age-matched general population group (5). The past year's (COVID-19 pandemic) dramatic changes resulted in increased stress, insecurity, fear of illness, risk of an imbalance between school-work and private life, and social isolation. Adolescents reply to these type of traumatic events with an increased incidence of anxiety-, eating disorders, depression, self-harm, suicide, and illegal substance use. A general increase in crime, where violence in close relationships and cyberbullying are some of the most frequent problems, is predicted. These are realities that high schools, social services, health cares, and families have to be prepared to face.

### Psychological/Behavioral Changes Induced by Yoga

In recent decades numerous research studies have investigated the effects and action mechanisms of yoga, often inclusive meditation and specific breathing techniques. Those studies suggest that such activities can have a significant impact on somatic and mental health. It has been shown that yoga increases psychological well-being, self-compassion, and resilience (6–8). For instance, some results suggest that yoga, including meditation and specific breathing techniques, can effectively reduce psychological distress (9, 10), anxiety, and depression (11–13). Yoga combined with controlled, same rhythm breathing was associated with a reduction in suicidal ideation for patients diagnosed with major depressive disorder (14). A specifically developed yoga intervention (trauma-informed yoga) has shown a reduction of symptoms in treatment-resistant PTSD (15).

There are also indications that yoga (including meditation and specific breathing techniques) can have beneficial effects on an individual's neurobiology and increase the capacity to regulate emotions by modulating the activity and connectivity in the prefrontal cortex, anterior cingulate cortex, insular cortex, and the amygdala (16, 17). Yoga improves the sympathetic nervous system's regulation and the hypothalamic-pituitary-adrenal system, which are essential in response to stress (18).

Today it is well-established that the environment (physical, psychological, social, and/or cultural) induces distinct gene expression changes, which can be studied by the emerging field of psychosocial genomics (19) focusing on the relationship between the body, mind, and behaviors. Studies on yoga and other mind-body interventions suggest that these practices positively affect gene expression profiles; for example, they may lead to a reduced risk of inflammation-related diseases (20). Multiple lines of evidence (preclinical, genetics, and bioinformatics) show an activation of immune system molecules and pathways that can contribute to psychiatric disorders' pathogenesis (21–23).

Last year a new review was published of randomized controlled trials testing the effects of yoga with youth (24), which concluded that there is growing evidence that yoga is a promising intervention for children and youth.

### Yoga in Correctional Settings—Our Previous Results

The above-described physiological changes may explain those behavioral and psychological changes observed in our previous study on yoga in the Swedish Prison and Probation Services (9, 25). In recent years, yoga has become popular in many correctional institutions worldwide as a complementary rehabilitation tool offered to inmates. The popularity can be partly explained by its cost and time efficacy compared to other non-pharmacological treatments, such as psychological therapies. Our study shows that regular yoga practice is associated with an increased level of impulse control (25), attenuation of aggression and antisocial behaviors (25), and a significantly decreased level of paranoid ideations (9), each of these phenomena being an essential variable related to criminal behavior. Furthermore, improvements on variables that can increase offenders' abilities to participate in treatments have also been observed, for example, that yoga significantly can increase positive emotional states and decrease emotional states that are negative (25), sustain attention (25), reduce depression, anxiety, and obsession (9), and very importantly, can increase personality traits, that can be interpreted as character maturity (26). Conclusively, our results, as well as other research summarized in a systematic review (27), show that yoga has a strong potential to strengthen the inmates' self-acceptance, purposefulness, and sense of responsibility. These are qualities that promote a more peaceful and safer environment in the correctional settings, providing a foundation for the development of a prosocial lifestyle upon release.

### Role of Yoga for Children and Young Adults at Juvenile Institutions

The overall goal of the treatments offered in SiS's juvenile institutions is to help adolescents adapt to social and cultural norms and become prosocial, healthy citizens. The adolescents' responsivity to such treatments is highly influenced by their sustained attention, compliance, and general mental health. Compulsory care providers experience a great need for new, cost, and time-effective non-pharmaceutical methods that can enhance the responsivity (attention, compliance) of young adults to the methods currently used to change antisocial behavior and treat substance-related syndromes. The most frequently used methods at juvenile institutions are Cognitive Behavioral Therapy, Aggression Replacement Treatment, Relapse Prevention, and Motivational Interview, each of which requires highly educated personal and longer treatment time. Importantly, we do not suggest yoga instead of any well-established treatment methods within SiS, but as a complement where yoga may promote and enhance the effects of ongoing psychological treatments. Yoga may also provide a positive coping tool for adolescents in SiS and potentially offer a prosocial activity upon release from institutions.

There is a lack of research measuring yoga's effects on adolescents' mental health, cognitive psychological characteristics, and deviant behaviors in residential/institutional care. One pilot study examined yoga's effects as a complementary care component in an institutionalized youth population (28). In that study, a multimodality intervention consisting of yoga poses, breathing exercises, and meditation was offered to institutionalized adolescents daily. The results indicate that yoga practice (combined with breathing techniques and relaxation/meditation) may improve stress resilience and self-control in an incarcerated youth population (28). In another pilot study, yoga intervention resulted in decreased alcohol consumption and improved social skills in a population of young people with high-risk behavior (29).

Contemplative preventative practices (manualized meditation-, mindfulness-based intervention) have been suggested in residential psychiatric care for children with severe psychiatric disabilities to reduce their need for physical intervention in response to dangerous behavior (30). A few studies have reported decreased anxiety and/or depression in children and adolescents (not institutionalized) having participated in yoga programs (31–35). It was proven that yoga and mindfulness-based interventions positively affect inattention and hyperactivity in young people with ADHD (36). Notably, a recent review of randomized controlled trials (RCTs) of yoga suggested improvement in behavioral and/or cognitive and/or physiological functioning in children and youth (ages 5–18; not institutionalized) (24).

## Methods

### Participants

Computer required sample size analysis for repeated measures ANOVA within-between interaction, with an effect size of 0.25 [medium effect size; average from effect sizes in changes by yoga in previous studies (9, 25)], a power of 0.80, an alpha error probability at 5%, for two groups (yoga and comparison) and two measurements (pre-and post-intervention) suggested a sample size of 34 for each sex.

We aim to collect complete data from at least 34 participants of both sexes (34 male and 34 female clients) to perform gender-specific analyses. Gender analyses will only be performed, if this number of participants can be reached.

Based on previous experience working with participants within correctional settings, we know that the attrition rate can reach 50%; therefore, our aim is to include approximately 120 participants.

**Figure 1:**
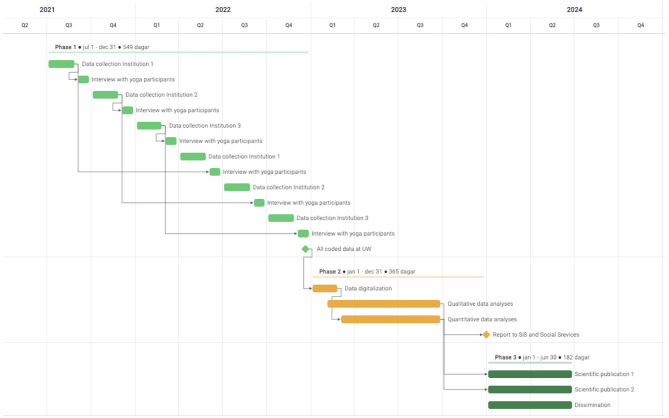
GANTT presentation of the project timeline.

Three institutions at the South West in Sweden have at this stage declared interest in participating in the study. Data collection is planned to take place for one and a half years' time, based on an estimated three months per institute (Figure 1). This three months period includes spreading advertisements and information about the study, collecting informed consents, six weeks of intervention (including pre- and post-intervention tests), and time for qualitative interviews to assess individual experiences of yoga classes. As the average time spent within SiS is calculated to be eight months, we will be able to perform intervention and data collection in each participating institute twice, six months apart during the planned data collection period (one and a half years) (Figure 1).

**Inclusion criterion:** All young people arriving at the juvenile institutions will be offered to participate in the study.
**Exclusion criteria:** Serious physical illness prohibiting participation in physical activities. Inability to read and understand at least one of the following languages: Swedish, Arabic, or Dari (variety of Persian spoken in Afghanistan).
**Inclusion criterion for the qualitative data collection:** Participation in the yoga intervention.
**Exclusion criteria for the qualitative data collection:** Inability to understand and speak Swedish language.

### Procedure

Upon placement to the juvenile institution, the young people will be offered to participate in the study. Participation is voluntary, and SiS clients are ensured that participation in yoga or in a comparison group or non-participation should not affect the care they receive at SiS. All data is coded (pseudo-anonymized) and the code keys will be kept securely at each juvenile institution under the institution directors' responsibility, following SiS regulations. Upon completion of data assessment code keys will be destroyed. Only anonym data will be handled outside each SiS facility. With this procedure we assure that there is no possibility of identifying the individual from the collected information.

**Figure 2:**
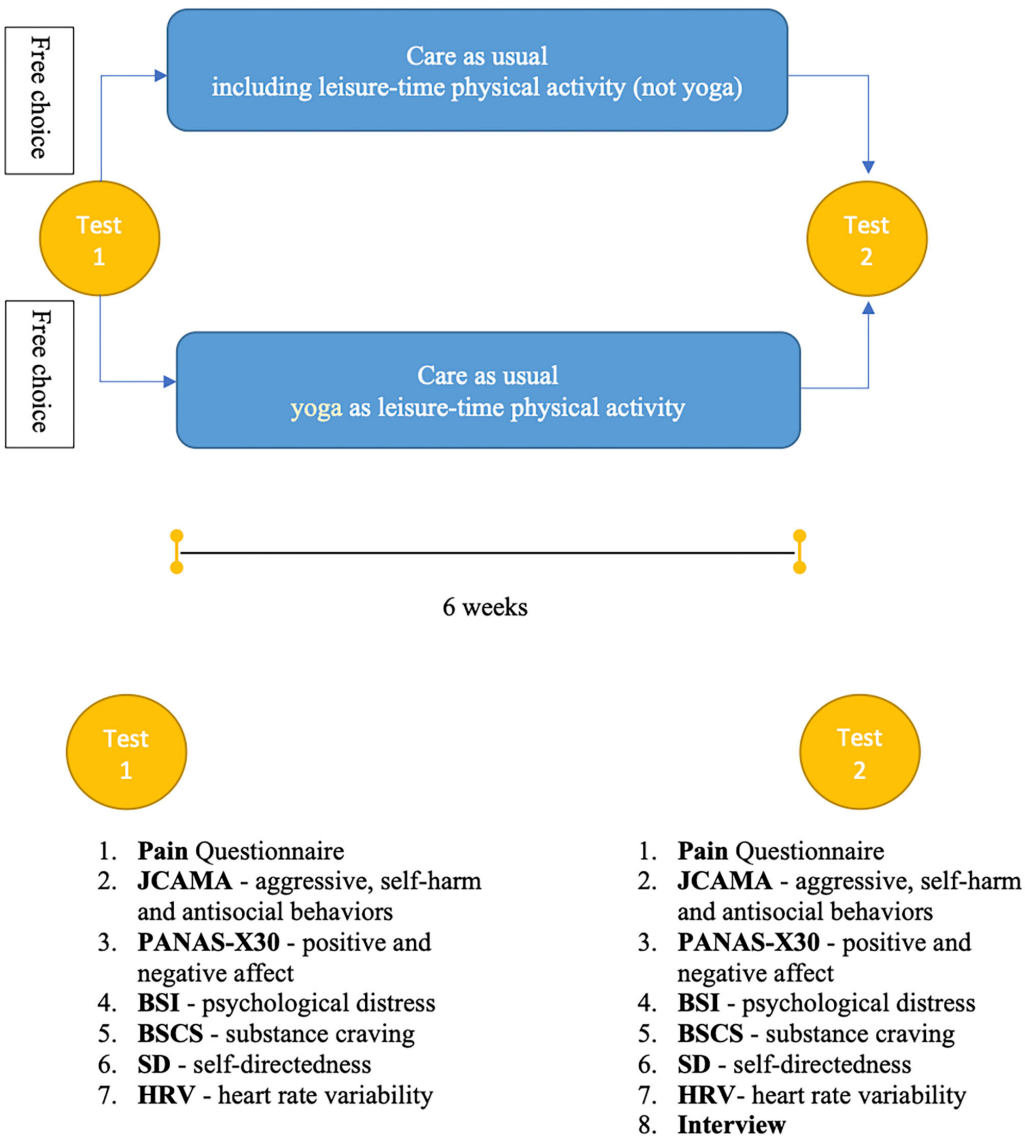
Flowchart of the quasi-experimental study design, and list of the included measures at pre- and post-intervention.

The study has a *quasi-experimental* design. Participants will receive a numeric code and will participate in the pre-intervention assessment, after which they will chose to participate in yoga classes (belonging to the “yoga group”) or not to participate in yoga classes (belonging to the “comparison group”) (Figure 2). The yoga group will participate in a 75 min yoga session, twice a week, for six weeks—as their leisure-time physical activity. During the same six-week period, the comparison group will participate twice a week (~1 h) in any, at the institution offered leisure-time physical activity (e.g., walking, gym training, and physical education class). Participants of the comparison group are asked to include ~10–15 min of relaxation at the end of their leisure-time physical activity.

#### Ethnicity

An essential aspect of our study is the inclusion of all major ethnic groups who are SiS clients. We have investigated how many languages spoken by most of the young people placed in SiS institutional care. As a result, we will offer the various test materials in Swedish, Arabic, and Dari, thus ensuring that almost 100% of the presumptive participants can take part in the study, independently of their ethnic belonging.

### Yoga Classes

The yoga classes held twice a week consist of trauma-informed yoga methods including inviting language, freedom of choice by offering options, a set routine for predictability and recognition, counting down in challenging poses for a sense of control and guidance toward practical interoceptive awareness. The relaxation offered is a progressive muscle relaxation adapted for ADHD and high anxiety levels. The aim is to increase resilience, promote relaxation and to provide self-regulation skills for stress and overwhelming emotions and reactions promoting self-empowerment and impulse control. The class will be set up in a circle to provide a sense of safety with no outer disturbances for triggering responses and will always be offered on a voluntary basis for the participants.

A yoga class is 75 min long and consists of yoga methods such as physical movement, static yoga postures, balance exercises, and breathing practices. The instruction intends to increase the interoceptive awareness with practically adapted mindfulness and guided trauma-informed progressive relaxation. The class includes intervals of raising and lowering heart rate and breathing to provide self-regulating skills. The classes do not consist of potential triggering poses, no physical adjustments or practices that can bear a resemblance to sexual abuse or practices that can over-activate the sympathetic nervous system.

Each class is built up as follows:

- Warm-up 15 min: At least 10 min of warm-up exercises to release muscular tension, discharge high-stress levels, restlessness, and anxiety, increase blood circulation, and increase proprioceptive awareness.
- Opening sequence 15 min: large energizing dynamic movements.
- Balancing poses 10 min.
- Dynamic Sequence 15 min: a combination between dynamic energizing movements and powerful postures where the participants hold the pose for at least two min (impulse-control). The importance of synchronized breathing and movements.
- Closing 20 min: including the last 10 min with guided mindfulness-based relaxation.

The instructors have completed a special training program in trauma-informed yoga and yoga for mental health, and they are trained in how to manage overwhelming emotions in a class. The classes are adapted to the most common mental health issues presented within the juvenile institutional system, such as PTSD, anxiety disorders, depression, ADHD, and autism.

The yoga manual is standardized, and all yoga instructors are qualified by a specific yoga-informed education course, held by licensed yoga therapist Josefin Wikström (e-RYT 500, YACEP, and TCTSY-F), founder of Trauma-adapted (informed) Yoga Sweden. She is the main teacher and Program director for the Prison Yoga Project Europe, recognized as a part of Trauma Research Foundation Therapeutic Alliance.

### Assessment

There will be two main testing periods: pre-intervention (Test 1) before the intervention and post-intervention (Test 2) after six weeks of intervention (Figure 2). All the measures described below will be included in the pre- and post-intervention tests.

### Measures

Three different origins of data are included as measurements: (A) self-reported measures in the form of a survey, (B) heart rate measure, and (C) register data. A and B applied both at pre-intervention (Test 1) and post-intervention (Test 2), while the data from SiS's register will be combined with data only for those participants who complete all parts of the study.

The pre-intervention survey includes a question on yoga expectations, and the post-intervention assessment includes a question on time spent on yoga and the experience of yoga.

#### A. Self-Reports

The self-report survey will include six previously validated instruments available in the Swedish, Arabic, and Dari languages.

##### Juvenile Care Adjusted Measure of Aggression

It is a SiS environment adapted version of the Prison Adjusted Measure of Aggression (PAMA), which assesses the self-reported frequency of state aggression and antisocial behavior in subjects within correctional settings (37). The Juvenile Care Adjusted Measure of Aggression (JCAMA) scale consists of nine items. The items are distributed over three subscales: a five-item Aggression scale, a two-item Antisocial Behavior scale, and a two-item Self-directed Aggression scale.

The Aggression subscale measures overt aggression and includes items on temper tantrums, verbal and indirect aggression, non-specific fighting, and physical assault against people. The Antisocial Behavior subscale consists of items on disciplinary problems at school and conflicts caused by own antisocial behavior. The Self-directed Aggression subscale includes items on self-injurious behavior and suicide attempts.

All the items are rated on a six-point scale based on the total number of occurrences over the past month: no occurrences = 0, one event = 1, two or three events = 2, four to nine events = 3, 10 or more events = 4, more events than can be counted = 5. The JCAMA score is the sum of the ratings on all nine items (total score) or the specific items in a certain subscale (subscale score). The JCAMA total score can range from 0 to 45; the Aggression subscale scores from 0 to 25; the Antisocial behavior scores from 0 to 10; and the Self-directed Aggression scores from 0 to 10.

The original PAMA's validity has previously been shown to be acceptable, and its test-retest reliability has been measured as fair (37), while the JCAMA reliability has not yet been tested.

##### Positive Affect and Negative Affect-Extended

The Positive Affect and Negative Affect-Extended (PANAS-X) instrument consists of 30 items and captures the valence (positive and negative) and the arousal (activated or deactivated) of the mood descriptors. As a result, PANAS-X30 makes it possible to measure positive (pleasant) and negative (unpleasant) and within both activated and deactivated affect states. This distinction is relevant in yoga studies, where effects are likely to be found in pleasant, deactivated emotions such as feeling calm, content, and relaxed. The following are examples for all four categories. Positive activated: *active, enthusiastic, excited, inspired* and, *proud*; Positive deactivated: *at ease, serene, calm, relaxed*, and *content*; Negative activated: *afraid, scared, hostile, guilty*, and *ashamed*; Negative deactivated: *tired, sluggish, drowsy, dull*, and *bored* (38).

The four scales' internal consistency is acceptable, with Cronbach's alphas ranging from 0.77 to 0.88 (25).

##### Brief Symptom Inventory

The Brief Symptom Inventory (BSI) is a self-rated measure of general psychopathology and psychological distress (39). The BSI contains 53 items reflecting psychological symptom patterns of psychiatric patients and non-patients. It comprises nine primary symptoms' scales and the global severity index (GSI). The primary symptom scales are: Anxiety, Depression, Interpersonal Sensitivity, Hostility, Obsessive Compulsiveness, Psychoticism, Paranoid Ideations, Phobic Anxiety, and Somatization. The answers are given on a five-point scale, from 0 = “not at all” to 4 = “extremely.” The BSI subscales and its GSI have high internal reliability (0.71–0.85) (9, 40, 41) and test-retest reliability, as well as high convergent, discriminant, and construct validity (42).

##### Pain

The Pain assessment instrument uses verbal and numerical assessment scales, where the participant describes their pain during the last two weeks using words (*no pain*; *mild pain*; *moderate pain*; *severe pain*; *very severe pain*; *unbearable pain*) and using numbers between 0 and 10, where 0 (zero) represents no pain, and 10 represents the worst possible pain. The type of pain and its location is marked by the participants on a standard image of a man or a woman (43).

##### Brief Substance Craving Scale

There have been multiple recommendations that craving should be included as a standardized outcome in treatment studies (44). The Brief Substance Craving Scale (BSCS), designed by Somoza et al. (45), will be used in the present study to measure the intensity, frequency, and lengths of cravings in the participants. BSCS measures craving for the primary substance, including alcohol and drugs. This short, self-report measure has previously been used and tested for psychometric properties (46, 47).

##### Self-Directedness

Self-directedness (SD) is a personality trait of self-determination. It captures the participant's ability to regulate, control, and adapt his/her behavior to situational requirements with the aim to achieve personally chosen goals. It is one dimension in Cloninger's Temperament and Character Inventory (TCI) (48). Cloninger has described SD as “willpower.” It captures to what extent a person identifies themself as an integrated, purposeful, self-sufficient, self-acceptant, responsible, reliable, and effective individual. SD contains 20 questions of the TCI-R140 scale.

##### Interviews

After the intervention (six weeks of yoga) qualitative information will be gathered about the participants' experience of yoga and possible health effects (please see the Supplementary Material for the semi-structured interview guide).

#### B. Heart Rate Variability

Heart rate variability (HRV), or the beat-to-beat alteration in heart rate, offers a noninvasive indicator of the autonomic nervous system's activity (49). HRV has been used as a proxy for health and fitness and as an indicator of autonomic regulation. Low HRV—indicating reduced parasympathetic cardiac control—was associated with sleep problems and difficulty regulating emotions (50), while yoga practice was associated with increased HRV (51).

HRV can be measured with a good quality activity bracelet and an App belonging to that, recording an electrocardiogram. At the moment we do not know which algorithmic approach for operationalizing HRV will be used. Data will be recorded during the first and last occasions of the intervention (yoga class). HRV data for the comparison group will be recorded with an identical time interval, during leisure time physical activity.

#### C. Register Data

Register data concerning the participants' medications during the intervention, their school attendance and participation in physical activity classes during the study will be assessed based on registers kept by SiS.

### Data Analysis

If the required number of male and female participants (at least 34 of each gender) is reached, then we will analyze the data separately by gender. Otherwise, the data will be pooled, and no gender-specific results will be presented.

All statistical analyses will be conducted using a significance level of *p* <0.05. Only complete post-attrition data will be analyzed. The comparison of the attrition rates between the two groups (“yoga” group and “comparison” group) will be calculated using Fisher's exact test. The Test 1 and 2 scores within the groups will be compared using the Wilcoxon signed-rank test. Mean changes and standard deviations will be calculated to identify differences within the groups. Mann–Whitney *U-*tests will be used for the continuous variables and Fisher's exact test for the categorical variables to compare differences between them. The effect sizes (Cohen's *d*) will be calculated to identify differences between the groups; an effect size of 0.2 will be considered a small effect, 0.5 a medium effect, and 0.8 a large effect. As data will be collected from three institutions, generalized estimating equations (GEE), that adjusts standard regression estimators for clustering, will be applied. The primary outcome is the level of psychological distress (BSI). Secondary outcomes' (craving, pain, affect, and aggressive antisocial behaviors) mediating and moderating effect will be also explored by conditional process analyses. We plan to conduct a mid-point analysis (about nine months after that data collection has started) to allow for better modeling of change trajectories.

To describe the adolescents' experiences of participating in yoga practice, with the help of a semi-structured interview guide (Supplementary Material), individual interviews will be performed. The adolescents will be contacted within 2 weeks after completing the yoga intervention. Content analysis will be used as a method for analyzing the interviews (52). The qualitative content analysis is performed stepwise identifying meaning units, condensing the meaning units, labeling meaning units with codes and thereafter identifying subcategories, categories ant themes. Content analysis makes it possible to describe the adolescents' experiences on both manifest and latent levels.

## Discussion

While there is a growing body of evidence in favor of the unique and significant positive effects of yoga as a form of complementary care for vulnerable populations (such as people with psychiatric conditions and/or people in correctional settings), there is a lack of research measuring the effects of yoga in the population of adolescents in residential/institutional care. The Swedish National Board of Institutional Care (Statens institutionsstyrelse, SiS) offers compulsory care for young people who manifest psychiatric disorders, substance use disorder and criminal behavior (2). The average age of an institutionalized young (fe)male is 16 years old, and (s)he meets the criteria for at least one psychiatric diagnosis. The most frequent diagnoses are ADHD, Substance Use Disorder, PTSD, Conduct Disorder, and Autism Spectrum Disorder. Depression and anxiety are usually co-existing diagnoses. One in four of these young people has attempted suicide. This population of adolescents has an increased need for effective treatments that can prevent the development of persistent mental ill-health, substance dependence, recidivistic criminal behavior, social marginalization, and untimely death. The young people's mental ill-health in SiS is substantial and extensive medical and psychological treatments are provided during their stay. SiS's goal and vision is, that the young people undergoing various SiS treatment programs should have gained by the end of their stay at SiS increased resources to live their lives without crime and abuse.

According to a follow-up study (5) today's institutional care is not sufficiently effective. Half of these young people have a history of at least one inpatient treatment episode, 60% are reconvicted for criminal acts, and 3% die within three years of their release from the institution.

Therefore, the planned study's impact can be measured from the aspects of the individual, the organizations, and the society.

Disruptive behaviors and high levels of psychological distress often hamper treatment responsivity. The suggested positive effects of yoga (decreased levels of psychological distress and less aggressive and antisocial behavior) may result in increased treatment responsivity, which may reduce the number of care days. This positive outcome may entail considerable benefits in terms of significantly decreased economic burdens on SiS and the society.

Reduced recidivism and substance use are not only valuable from a socioeconomic perspective, but they are also necessary for the promotion of a safe society for all and, of course, represent a vital benefit for the individual. The study population comprises a highly relevant group from the perspective of basic societal needs. There are about 550,000 15–19-year-old Swedish citizens today. 0.2% of these adolescents are institutionalized in SiS care yearly (4). The care provided to this small fraction of adolescents represents a considerable annual cost for society (1.6 million SEK/person for medication and 2.1 million SEK/person for placement in SiS care). If this vulnerable group's care and rehabilitation are not successful, it will continue to represent a considerable burden on society at several levels: criminality, medical health problems, social support needs, pain, and suffering of the individual and others.

An active heroin addict costs society 2.1 million and an amphetamine addict about 1.5 million SEK/year from an economic perspective. These figures encompass the expenses for their active use (criminality, legal authorities, and financial aid), medical and social care, and costs for their family's support.

In the event the present study can provide evidence on the positive effects of yoga for the clients of SiS institutions, it will provide a basis and rationale for making a strategic investment at the national level.

The positive effects of yoga at the individual level are proposed from strong scientific evidence. Studies have proven in adult populations that yoga can strengthen the practitioner's self-acceptance, purposefulness, and sense of responsibility (26). These qualities promote a more peaceful and safer environment in institutional settings and offer higher effectiveness of other treatments and school education programs. Yoga can also provide a foundation for the development of a prosocial lifestyle upon release. In other words, there is evidence that yoga empowers the individual yoga practitioner and thus improves his or her mental health and prosocial lifestyle. These novel effects of yoga practice emphasize the importance of testing yoga as complementary care for young people with behavioral and mental health problems.

The duration and the type of yoga in the present study have been adjusted to the population. We use a shorter intervention time but more frequent training (six weeks twice a week, implying 12 yoga sessions) to optimally adapt to the participating institutes' possibilities. Several studies have proven that 8–10 weeks (once a week, therefore, 8–10 sessions) yoga has positive effects on mental health, behaviors, and self-control of adults in correctional setting (9, 25–27, 53). As our study population includes adolescent, it is essential to recognize that the brain goes through pruning (anatomically improving its efficiency) during this developmental period [see e.g., (54)]. During this time, the brain is increasingly susceptible to environmental effects; therefore, we speculate that eventual changes in adolescents' mental health and behaviors will be recognizable already after six weeks (12 sessions) of training. There are results indicating that short-term intensive interventions (four weeks, three times a week yoga classes) have a positive effect on adults' anxiety, depression, and stress (55). Importantly we use a specifically developed yoga (trauma-informed yoga), adapted to the most common psychiatric disorders (ADHD, PTSD, anxiety, and depression) in the adolescent study population.

The proposed study fills a gap in knowledge focusing on young people population in institutional care. If the study's hypothesis is confirmed, our results will verify and strengthen earlier findings in Sweden (9, 25, 26) regarding the benefits of yoga for a marginalized population, thus indicating the usefulness and value of yoga within juvenile institutional care at a national level.

One limitation of the proposed study is the fact that we won't be able to randomly distribute participants between groups, with other words, we could not rationalize a randomized controlled trial (RCT) design. A quasi-experimental design was chosen in agreement with the clinical and institutional experts, who predicted an unacceptable high attrition rate (80–90%) in case of not giving the possibility to the young clients to freely choose their participation in yoga classes.

Future RCTs should confirm our findings and prospective longitudinal studies need to investigate if yoga within SiS can potentially offer a prosocial activity upon release from institutions for young people.

### Ethics and Dissemination

Young people placed in SiS institutions are in many respects in a vulnerable position because of their deprived situation. From an ethical research perspective, therefore, it should be taken into account that this position may affect their propensity to decline to participate in the study due to fear of negative consequences. However, this risk may be considered small. Therefore, great emphasis will be placed on the research ethical principles regarding information, consent, confidentiality, and usefulness. Consequently, all participants will be informed, both orally and in writing, about the purpose and structure of the study, that participation is voluntary, and that they have the right to end their participation at any time. Written consent will be obtained from all study participants before inclusion in the study. All data and information gathered will be treated with the highest possible confidentiality. In this context, it should also be clear from the information that the decision to participate or not in the study, will not affect the young person's treatment plan in any way.

The National Ethical Review Authority has approved the study with the registration number: 2019-04589. We applied for a change in the original ethical approval as we have changed—based on last year's feedback on our grant applications—the study design from RCT to quasi-experimental design, and we included qualitative interviews in the project. The new ethical approval is registered with Dnr: 2021-00088.

This project is carried out in close collaboration with the Swedish National Board of Institutional Care (Statens institutionsstyrelse, SiS), an independent Swedish government agency operating a multidisciplinary activity. The care and treatments offered within SiS institutions must be based on the individual's needs (person-centered care). On a national level, only methods for which there is scientific support are used. Yoga is offered already in some SiS institutions, but not included in care, because of the lack of research and evidence.

The project will take part in University West's academic environment, where the (in Sweden) unique Social Psychiatric Care candidate program is established. The Social Psychiatric Care program covers the interfaces between social services, psychiatric healthcare, the police, correctional centers, schools, and non-governmental organizations. The project is also coupled to the research center for Child and Youth Studies at University West (56), which is part of a network of similar research environments at Stockholm University, Linköping University, University of Gothenburg, and Malmö University. The purpose of the network is to gather and promote research in the interdisciplinary field of child and youth studies.

The project is supported by the Prison Yoga Project (PYP) (57), a non-profit organization that supports incarcerated people worldwide with trauma-informed yoga and mindfulness programs. The PYP Community includes a wide range of professionals (academics, certified yoga instructors, prison employees, medical doctors, etc.) with a common aim to promote social change, transforming the systems and culture to create a more inclusive, equitable, and just world.

Information about the project is disseminated using several channels: producing reports to SiS, open accesses peer-reviewed scientific articles, giving scientific and popular scientific lectures at national and international forums, and using the above-described collaborations.

## Ethics Statement

The National Ethical Review Authority has approved the study with the registration numbers: 2019-04589 and 2021-00088. Written informed consent to participate in this study will be provided of the legal guardian (in case of the participants is under the age of 15) and all the partcipants.

## Author Contributions

NK is the project leader who has planned the study and developed the study protocol in cooperation with national agents and SiS employers.

## Conflict of Interest

The author declares that the research was conducted in the absence of any commercial or financial relationships that could be construed as a potential conflict of interest.
