# Supplementary material for: Yoga as Complementary Care for Young People Placed in Juvenile Institutions—A Study Plan
Source: Front Psychiatry. 2021 Jun 4;12:575147. doi: 10.3389/fpsyt.2021.575147 (PMC8211756; doi:10.3389/fpsyt.2021.575147)
Supplement: Supplementary file 1 [file Table_1.pdf]

Semi-structured interview guide to the study
”Yoga as complementary care for young people placed in juvenile institutions.”

Today, I will be asking you about your experience practicing yoga at the institution over the past six weeks. Please remember that there are no right or wrong answers. I want to hear your true opinion and your perspective.

1. What did you think the yoga classes would be like?

    - Were there any ways that the yoga classes were different than what you expected?
    - In what way did you think the yoga classes might be helpful to you?
    - Did you have any worries about the yoga classes? What were they?

2. Please describe your experience participating in the yoga classes.

    - How did participating in yoga impact your physical health?
    - How did participating in yoga impact your mental health?
    - What parts did you like best about the yoga classes?
    - What parts of the yoga classes did you dislike?

3. What did you learn—if anything—about yourself by participating in the yoga classes?

4. In what ways do you think---if any—participating in the yoga classes affect your ability to manage your mindset, or your thoughts?

    - How did yoga affect the way you thought about things when you were scared or when you experienced a stressful or unexpected event?

5. In what ways--if any—did participating in the yoga classes affect your ability to manage your emotions?

    - How did participating in yoga impact your mood?
    - Hur påverkades ditt stämningsläge av att delta i yoga?

6. In what ways—if any— did participating in the yoga classes affect your behavior?

    - How did participating in yoga impact what you did when you experienced anger, sadness or stress?

7. What were the things—if any—that were said in the yoga classes that stuck with you the most?

8. What are your thoughts about yoga compared to the other leisure time physical activities that you participated in during your placement?

    - What similarities and what differences you would name between yoga and other leisure time physical activities?

9. In what ways—if any—do you think you can apply what you learned in the yoga classes in the future?

10. Can you imagine continuing to attend yoga classes in the future?

11. What are your recommendations for future classes?

Thank you for your time and participation!
